# Editorial: Thyroid Hormone and Metabolites: Central Versus Peripheral Effects

**DOI:** 10.3389/fendo.2019.00240

**Published:** 2019-04-16

**Authors:** Grazia Chiellini, Federica Cioffi, Rosalba Senese, Pieter de Lange

**Affiliations:** ^1^Dipartimento di Patologia Chirurgica, Medica, Molecolare e dell'Area Critica, Università degli Studi di Pisa, Pisa, Italy; ^2^Dipartimento di Scienze e Tecnologie, Università degli Studi del Sannio, Benevento, Italy; ^3^Dipartimento di Scienze e Tecnologie Ambientali, Biologiche e Farmaceutiche, Università degli Studi della Campania “Luigi Vanvitelli”, Caserta, Italy

**Keywords:** 3,5,3′-triiodo-L-thyronine, 3,5-diiodo-L-thyronine, 3-iodothyronamine, lipid metabolism, glucose metabolism, thermogenesis, bone remodeling, behavior

The organism's metabolic state is under tight control and an efficient balance between glucose and lipid metabolism is crucial for maintenance of energy homeostasis, which allows the body to function optimally. Metabolic homeostasis is under control of hormones as well as the sympathetic/parasympathetic nervous systems. Particularly thyroid hormone (TH) plays an important role in the regulation of energy expenditure. The action of metabolically active TH metabolites involves a plethora of avenues which have been and are still being unraveled from the previous century to the present day. At the cellular level, the classic, biologically active TH, 3,5,3′-triiodo-L-thyronine (T3), predominantly acts through thyroid hormone receptors (THRs), which are predominantly localized in the nucleus and, upon binding of T3, act as ligand-dependent transcription factors that regulate gene transcription through binding to thyroid hormone response elements (TREs). The THRs, though, are also known to act within the cytoplasm. In addition, T3 acts at the cell membrane level. All these effects, which are either “genomic” (involving gene transcription) or “non-genomic” (involving activation of pathways through resident proteins in the cell) are mediated directly by TH within the cell and are referred to in this Topic as “peripheral effects.” In addition, as highlighted in this Research Topic, thyroid hormones can activate the central and autonomic nervous system, which, by acting through their appropriate membrane receptors, regulate cellular responses, not necessarily involving the intracellular response to the hormone itself. The TH-mediated effects of the autonomous and central nervous system are referred to in this Topic as “central” effects. This intriguing issue is presented and discussed here in 3 original research papers, 3 reviews, and 4 minireviews, in which the central as well as peripheral effects are highlighted. They address the mechanisms of action of the metabolically active THs T3, 3,5-diiodo-L-thyronine (T2), 3-iodothyronamine (T1AM), and their action on various tissues including liver, skeletal muscle, heart, bone, brain, and white and brown adipose tissue. Although the field is in rapid progress, the contributions successfully cover the current state-of-the-art and clearly present the vast versatility of action of hormones which directly or indirectly originate from the thyroid gland. A clear example of the combined action of T3 on the nervous system and the target cell itself is the induction of thermogenesis in brown adipose tissue, elegantly reviewed by Cioffi et al. BAT thermogenesis requires increased transcription of both uncoupling protein 1 (UCP1), inducing dissipation of energy through heat at the mitochondrial level, and of peroxisome proliferator-activated receptor γ coactivator-1α (PGC-1α), triggering transcription of genes involved in mitochondrial duplication and thermogenesis. UCP1 transcription is under direct control of T3 within the brown adipocyte through THR beta, whereas PGC-1alpha expression is under control of Norepinephrine (NE), a neurotransmitter of the sympathetic nervous system (SNS). Direct proof that both the nervous and the intracellular response are under control of T3 came from studies showing that T3 triggers SNS-mediated BAT activation upon hypothalamic injection. Since it has recently been shown that T2, one metabolically active thyroid hormone metabolite, is also able to activate BAT associated with increased UCP1 activity and NE-mediated signaling, a currently unresolved question is whether the effect of T3 on BAT is partly due to the conversion of T3 into T2. Interestingly, using the same technique revealed that T3, by interacting with the parasympathetic nervous system (PSNS), induces hepatic lipogenesis through de-phosphorylation of the AMP-activated protein kinase (AMPK), key to the induction of fatty acid oxidation in target tissues. Further evidence for central effects of T3 comes from an interesting study by Martins et al. showing that T3 acts on the SNS to control bone remodeling. Treatment of mice with depletions of the α2A or α2C adrenoreceptors with a supraphysiological dose of T3 alleviates the negative effects of the resulting thyrotoxicosis on distinct bone compartments. Also the central nervous system (CNS) is a target of TH metabolites. As reviewed by Laurino et al. T1AM stimulates metabolism and behavior in rodents, and its levels are critically regulated in the brain. Behavioral effects related to T1AM in the brain presumably involve trace amine-associated receptor 1, the histaminergic system, and mitochondrial monoamine oxidases. Peripheral effects of TH on metabolism in different contexts are reviewed by Kowalik et al.; Duntas and Brenta, and Louzada and Carvalho. Kowalik et al. highlight the effect of thyromimetics vs. TH metabolites on liver diseases ranging from non-alcoholic fatty liver disease to hepatocellular carcinoma in cell and rodent models, whereas Duntas and Brenta focus on lipid metabolism by TH in humans, and Louzada and Carvalho discuss to what extent the effects of TH metabolites mechanistically overlap in metabolically active tissues. Cicatiello et al. review why local conversion of T4 into T3 by type 1 and type 2 iodothyronine deiodinases, and TH inactivation type 3 iodothyronine deiodinase, is of crucial importance to render metabolically active tissues capable to promptly respond to various metabolic demands posed by for instance cold exposure or physical exercise without perturbing systemic TH levels. One TH metabolite, T2, mainly acts through THR-independent ways, as reviewed by Senese et al. who further highlight that in rodent diet-induced obesity models, T2 restores insulin sensitivity in skeletal muscle, a crucial tissue for the maintenance of normoglycemia. In this light, the study by Sacripanti et al. elegantly shows that in the isolated rat heart perfused with low concentrations of T2 (0.1 or 1.0 mM) significant increases in glucose uptake are observed (by 24 and 35%, respectively), without an alteration of cardiac output. Since T3 and T4 did not increase glucose consumption, direct cellular action of T2 may proof promising in increasing the response to glucose and, eventually, the efficient management of disturbances in glycemia related to diabetes type 2. Finally, Lorenzini et al. present an elegant method based on mass spectrometry (MS) that enables to precisely quantify 3,5-T2 and 3,3′-T2 in human serum. Overall, the original articles and reviews of this research topic clearly demonstrate that thyroid hormone action not only comprises an intricate network of distinct cellular pathways, differentially activated by each metabolite present in the cell, but also nervous signaling, which involves both the CNS, the PSNS, and the SNS. All these pathways converge to provide the final metabolic phenotype dictated by the thyroid. Future research is necessary to unravel these pathways, which will, as we hope, be triggered based on the information provided by the contributions to this research topic, and the relevant literature cited therein.

## Author Contributions

GC, FC, and RS: contributions to the conception of the work; PdL: contribution in drafting of the work and of the final approval of the version to be published.

### Conflict of Interest Statement

The authors declare that the research was conducted in the absence of any commercial or financial relationships that could be construed as a potential conflict of interest.

